# FOXP3^+^ Cells in Tertiary Lymphoid Structures Have Adverse Impact on Overall Survival in Patients with Gastric Cancer

**DOI:** 10.3390/medsci14010145

**Published:** 2026-03-18

**Authors:** Ana Paparella Karaman, Tomislav Ivanović, Krešimir Mustapić, Katarina Vukojević, Luka Minarik, Merica Glavina Durdov, Petar Đolonga

**Affiliations:** 1 Department of Pathology, Forensic Medicine and Cytology, University Hospital of Split, 21000 Split, Croatia; ana.paparella-karama@kbsplit.hr (A.P.K.); merigdst@yahoo.co.uk (M.G.D.); 2 Department of Surgery, University Hospital of Split, 21000 Split, Croatia; tomo.mefst@gmail.com (T.I.); kresimir.mustapic@gmail.com (K.M.); 3 Department of Anatomy, Histology and Embryology, University of Split School of Medicine, 21000 Split, Croatia; katarina.vukojevic@mefst.hr; 4 Department of ENT, Čakovec County Hospital, 40000 Čakovec, Croatia

**Keywords:** gastric cancer, gastrectomy, tertiary lymphoid structures, FOXP3^+^ regulatory T lymphocytes, outcome

## Abstract

**Background/Objectives**: Patients with local/locally advanced gastric cancer (GC) undergo gastrectomy/lymphadenectomy, but recurrences are common and the disease usually progresses to death. Tertiary lymphoid structures (TLS) of varying maturity can be observed in the immune microenvironment of the primary tumor. The aim of the study was to analyze the association of TLSs and their immune cellular composition with clinicopathological variables and overall survival (OS). **Methods**: In a cohort of 92 GC patients who underwent gastrectomy, the characteristics of tumor core TLSs were assessed and the density of cytotoxic CD8^+^ T cells and regulatory FOXP3^+^ T cells was analyzed. **Results**: Patients with TLS had a better OS than patients without TLS, 19.4 months vs. 9.2 months (*p* = 0.001). Immature TLSs were more frequently associated with lymphovascular invasion and regional lymph node metastasis (*p* = 0.014 and *p* = 0.034). Mature TLSs had a higher FOXP3^+^ T lymphocyte density and lower CD8^+^/FOXP3^+^ ratio than immature TLSs (*p* = 0.029 and *p* = 0.013), and patients had a longer OS than patients with immature TLSs, 34.55 months vs. 15.2 months (*p* = 0.033). In patients with TLS-positive GC, cases with FOXP3^+^ T cells had a shorter OS, 12.7 months vs. 47.5 months (*p* < 0.001). **Conclusions**: The presence of FOXP3^+^ cells in TLS is associated with significantly shorter OS of patients with local/locally advanced GC.

## 1. Introduction

Gastric cancer (GC) remains among the five leading causes of cancer-related morbidity and mortality worldwide, representing a major public health challenge [1]. Despite significant advances in diagnostics and treatment, patient prognosis continues to be poor, largely due to late diagnosis and biological heterogeneity [2,3]. Although the tumor-node-metastasis (TNM) classification remains the gold standard for GC staging, additional prognostic criteria could be valuable, particularly considering the variability in clinical outcomes even among patients classified within the same TNM category [4]. Traditionally, gastric adenocarcinoma has been classified according to the Lauren classification into intestinal and diffuse types [5], while the World Health Organization (WHO) classification further distinguishes several histological variants [6]. Moreover, WHO even suggests further molecular subtyping into four categories: Epstein–Barr virus (EBV)-positive, microsatellite instability-high, genomically stable, and chromosomal instability types [7]. This molecular framework complements morphology-based systems and offers potential for refined prognostic assessment and personalized therapy [8].

Incorporating additional parameters such as tumor budding, tumor–stroma ratio, HER2 expression, claudin-4 status, and Stroma A-Reactive Invasion Front Areas (SARIFA) could enhance prognostic accuracy and support individualized treatment strategies [7,9,10,11].

Over the past decade, the tumor immune microenvironment (TIM) has been recognized as a crucial determinant of tumor progression and patient outcome [12,13]. Accordingly, there is increasing interest in understanding the mechanisms that enable an effective antitumor immune response, including the role of tertiary lymphoid structures (TLSs) [2]. TLSs are ectopic, organized, non-encapsulated aggregates of immune cells that develop within non-lymphoid tissues in response to persistent pathological stimuli, such as infection, chronic inflammation or malignancy. Identified in various non-lymphoid organs, TLSs structurally and developmentally resemble secondary lymphoid organs, but exhibit different degrees of organization, ranging from immature lymphoid aggregates to mature, well-structured formations [2,4,14]. TLSs are mainly composed of B and T lymphocytes, follicular dendritic cells, and high endothelial venules [14,15,16]. Several studies have reported that the presence of TLSs within the TIM correlates with better prognosis in various malignancies, including gastric cancer [2,9,10,11]. Nevertheless, conflicting evidence also exists, suggesting that their prognostic role may vary depending on tumor type, localization, and immune composition [3,4,17]. The most available data originate from studies conducted in Eastern populations, while information on Western cohorts remains limited [4]. The aim of this study was to elucidate the prognostic significance of TLSs in a cohort of patients from a single institution in Southern Croatia.

## 2. Materials and Methods

### 2.1. Patient Selection

This retrospective study included 92 patients with GC who underwent total gastrectomy and lymphadenectomy at the Department of Surgery, University Hospital of Split between 2015 and 2019. Clinicopathological data: age, sex, histological subtype of GC, lymphovascular and perineural invasion, and pathological stage, were collected from pathology reports. Exclusion criteria were lack of clinical follow-up and previous neoadjuvant therapy. Data were obtained from hospital registries and a national mortality database. OS was defined as the interval between the date of pathological diagnosis and death from any cause or the last day of follow-up on 31 May 2025.

Representative paraffin blocks were retrieved from the archives of the Department of Pathology, Forensic Medicine and Cytology, University Hospital Split and stained with standard H&E staining, and immunohistochemically with primary antibodies CD8^+^ and FOXP3^+^ (both Ventana, Tucson, AZ, USA) on immunostainer Ultra benchmark (Ventana, Tucson, AZ, USA).

### 2.2. Evaluation of Size and Maturity of Tertiary Lymphoid Structures

**Figure 1:**
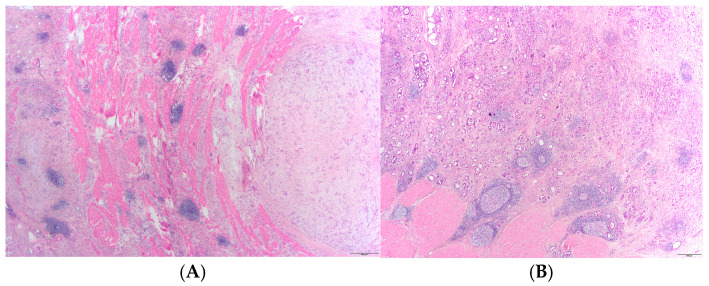
Tertiary lymphoid structures in gastric adenocarcinoma: immature (**A**) and mature (**B**) with germinal centers (20×).

Representative histological slides of tumor tissue were examined under a light microscope (Olympus 63, Okazaki, Japan), photographed with a digital camera (Olympus, Okazaki, Japan) and analyzed using Cell Sens software (Olympus, Okazaki, Japan). TLS is defined as an unencapsulated organized lymphoid aggregate at least 200 µm in diameter, located in the mucosa, tumor itself, or at the tumor margin [4]. TLS density was determined by the number of TLSs in 3 consecutive microscopic fields at 40× magnification (the area of one field was approximately 9.0 mm^2^). All sections were independently analyzed by two observers (APK, MGD), and any discrepancies were resolved by consensus review. The average TLS area (mm^2^) was calculated as the mean of 4 consecutive TLSs on the slide. Mature TLSs were defined as well-organized lymphoid structures containing B-cell follicles with a germinal center, while immature TLSs consisted of loosely arranged aggregates of lymphocytes without recognizable follicular organization [18,19]. Lymphoid aggregates are classified as immature TLSs [19,20]. Cases with only immature TLSs were classified as immature, whereas cases containing mature TLSs or a mixture of mature and immature TLSs were classified as mature (Figure 1).

### 2.3. Immune Cell Density in Tertiary Lymphoid Structures

**Figure 2:**
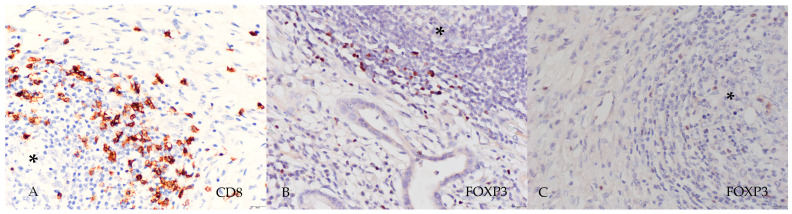
Immunohistochemical staining of CD8^+^ T cells (**A**) and FOXP3^+^ regulatory T cells in tertiary lymphoid structures (*) observed in intestinal (**B**) and diffuse (**C**) subtypes of gastric adenocarcinoma (400×).

The slides were microscopically analyzed and photographed at 400× magnification (Figure 2). The number of lymphocytes positive for CD8^+^ (brown membranous staining) and FOXP3^+^ (brown nuclear staining) was counted using CellSens software v. 1.17 and expressed as the absolute number of positive cells per mean TLS area (number of cells/mm^2^).

### 2.4. Transcriptomic Data

To further evaluate the transcriptomic expression of FOXP3, molecular and clinical data were obtained from The Cancer Genome Atlas (TCGA). The dataset was accessed via the cBioPortal platform (https://www.cbioportal.org) on 5 March 2026, using the STAD TCGA PanCancer Atlas dataset. FOXP3 mRNA expression values (z-scores) were extracted and matched with corresponding clinical data using unique patient identifiers. Overall survival (OS) was defined as the time from diagnosis to death or last follow-up and was expressed in months.

### 2.5. Statistical Analysis

Categorical data are presented as absolute frequencies (*n*) and percentages (%). Differences between categorical variables were determined using the chi-square test. For numerical data, the normality of distribution was tested using the Shapiro–Wilk test. Due to significant deviation from normality, numerical data were expressed as median and interquartile range (IQR). Significance of differences between groups was established using the Mann–Whitney U test. Survival analysis was performed by the Kaplan–Meier method and multivariable Cox proportional hazards regression. A *p*-value of <0.05 was considered statistically significant. All statistical analyses were performed using IBM SPSS Statistics for Windows, Version 26 (IBM Corp., Armonk, NY, USA), and RStudio software (v. 2024.12.1+563).

## 3. Results

### 3.1. Clinicopathological Characteristics of the Study Cohort

The study included 92 patients with local/locally advanced GC, 63 (68.5%) male and 29 (31.5%) female. The median age was 75 years (IQR: 64–81). Intestinal-type adenocarcinoma predominated (63.3%). According to Bormann, ulcerative-vegetative (27.5%) and ulcerative (33.0%) types were the most frequent, followed by infiltrative (23.1%) and polypoid (16.5%) types. Most tumors were classified as pT3–4 (79.3%) with regional lymph node metastases in 73.9% of cases. Lymphovascular invasion was present in 78.6%, and perineural invasion in 62.3% of cases. TLSs were identified in 66 cases (71.7%), whereas 26 tumors (28.3%) lacked TLS formation.

When patients were stratified according to the presence of TLSs (Table 1), no statistically significant differences were observed between the groups in terms of sex, age, histological or macroscopic type, depth of invasion, lymph node involvement, or the presence of lymphovascular and perineural invasion. However, patients with TLS-positive tumors had a significantly longer overall survival (OS) than patients with TLS-negative tumors (*p* = 0.006).

### 3.2. Survival Analysis According to the Presence of TLSs

Kaplan–Meier survival analysis was performed on 85 patients, with follow-up from the time of diagnosis to death or censoring in June 2025. A total of 9 patients (10.6%) were censored, and 7 patients were excluded from the analysis due to missing survival data. The median survival time was 13.5 months (95% CI: 7.979–19.021). Median overall survival times stratified by clinical stage were calculated and are presented in Supplementary Table S1.

**Figure 3:**
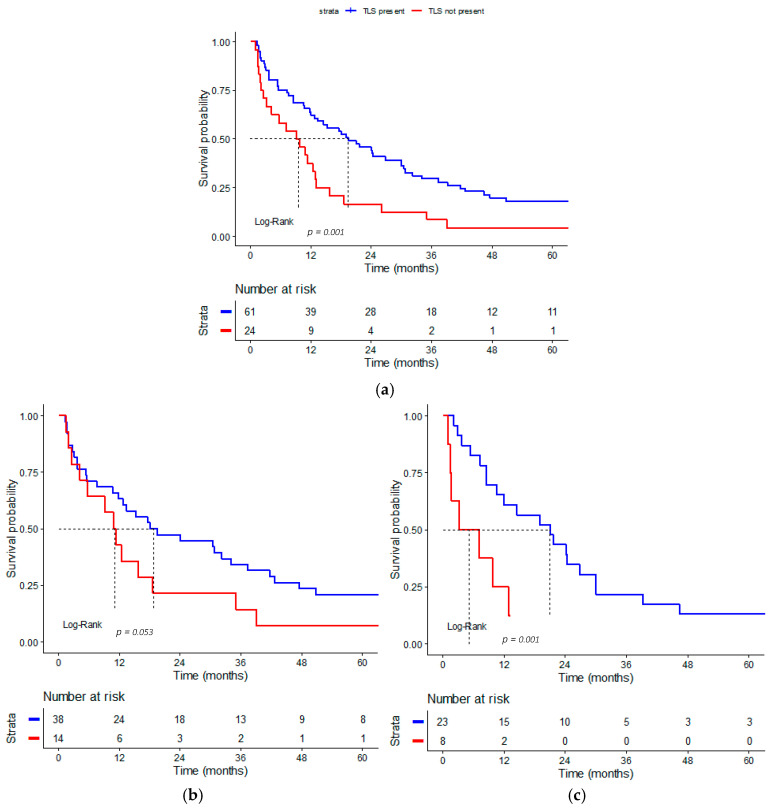
Kaplan–Meier survival curves according to TLS status: (**a**) all patients; (**b**) patients with intestinal-type GC; (**c**) patients with diffuse/mixed-type GC. The dashed lines represent the median survival time.

There was a statistically significant difference in OS between patients with and without intratumoral TLS (Figure 3a). A significant difference in 2-year survival was found between patients with TLS-positive and TLS-negative tumors. Median survival of patients who had TLS-positive tumors was 19.4 months (95% CI: 9.779–29.021), and 9.2 months (95% CI: 3.079–15.321) in patients with TLS-negative tumors (Log-Rank test, *p* = 0.001). The estimated two-year survival rate was 44.3% (95% CI: 33.4–58.7) for TLS-positive patients and 16.7% (95% CI: 6.8–40.8) for TLS-negative patients.

When analyzed by Lauren histological subtype (Figure 3b,c), similar survival trends were observed. In intestinal-type tumors, median survival time was 15.218 months (95% CI: 8.604–21.796) in TLS-positive cases and 10.8 months (95% CI: 6.950–14.650) in TLS-negative cases (Log-Rank test, *p* = 0.053). In diffuse/mixed tumors, median survival reached 21 months (95% CI: 9.887–32.113) versus 3.3 months (95% CI: 0–10.784) in TLS-negative cases (Log-Rank test, *p* = 0.001).

Multivariable Cox regression was performed, adjusted for age, pathological stage (pT), nodal status, and Lauren subtype (Table 2). The presence of TLS remained an independent predictor of improved OS (HR 0.497, 95% CI 0.281–0.88, *p* = 0.017).

### 3.3. Clinicopathological Variables in Relation to the Number and Maturity of TLSs

Clinicopathological features and immune cell densities in TLSs were analyzed in relation to TLS maturation (Table 3). Most TLSs were classified as lymphoid aggregates or non-mature forms (49, 74.2%), while 17 (25.8%) were classified as mature. Samples with mature TLSs exhibited a higher number of TLSs (*p* = 0.001) and larger dimensions of individual TLSs (*p* = 0.016). Immature forms of TLSs were associated with more prevalent lymphovascular invasion (ꭓ^2^ = 6.081, *p* = 0.014) and more frequently exhibited lymph node metastases (ꭓ^2^ = 4.520, *p* = 0.034). On the other hand, mature TLSs demonstrated higher density of regulatory FOXP3^+^ T lymphocytes (*p* = 0.029) and a lower CD8^+^/FOXP3^+^ ratio (*p* = 0.013), while CD8^+^ lymphocyte density did not differ significantly between groups (*p* = 0.795). Although there was no significant difference in distribution of pathological and clinical stages between the groups, patients with mature forms reached better OS than those with immature forms of TLSs, 34.55 months (IQR: 18.93–87.03) versus 15.2 months (IQR: 5.4–34) (*p* = 0.033).

### 3.4. Impact of FOXP3^+^ Regulatory T Lymphocytes in TLSs on Patients’ Survival

**Figure 4:**
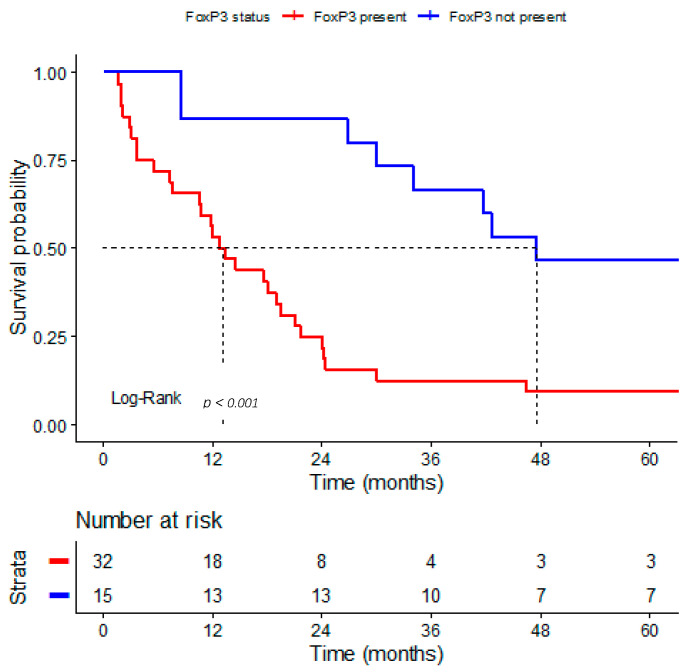
Kaplan–Meier survival analysis according to the presence of FOXP3^+^ regulatory T cells in TLSs in gastric adenocarcinoma. The dashed lines represent the median survival time.

In 47 patients, survival analysis was performed in relation to regulatory FOXP3^+^ T lymphocytes in intratumoral TLSs (Figure 4). OS was significantly shorter in patients who had regulatory FOXP3^+^ T lymphocytes in TLSs (median 12.7 months, 95% CI: 8.958–16.442) compared to those without these cells (median 47.5 months, 95% CI: 0–101.655) (Log-Rank test, *p* < 0.001). The estimated 2-year survival rate was 21.9% (95% CI: 11.4–42.1) for patients with FOXP3^+^ regulatory T cells in TLSs and 86.7% (95% CI: 71–100) for patients without them.

Multivariable Cox regression was performed, adjusted for age, pathological stage (pT), and nodal status (Table 4). The presence of regulatory T cells in TLSs remained an independent predictor of worse OS (HR 8.165, 95% CI 3.341–19.955, *p* < 0.001).

### 3.5. Transcriptomic Data

Analysis of transcriptomic data from the TCGA cohort demonstrated that patients with higher FOXP3 expression did not exhibit longer OS compared with patients with lower FOXP3 expression (log-rank test, *p* = 0.48) (Supplementary Figure S1).

## 4. Discussion

The tumor microenvironment interacts with tumor cells, shaping tumor biological behavior, proliferation, angiogenesis, and invasiveness, while modulating the host immune response [21]. In GC, the microenvironment plays a key role in establishing an immunosuppressive milieu [22] that supports tumor progression and resistance to therapy, but can also promote the development of TLSs as a sign of antitumor immunity activation [2]. Chronic antigenic stimulation recruits antigen-presenting cells and lymphocytes, leading to their accumulation within the tumor [23,24,25,26,27]. Activated fibroblasts acquire a phenotype resembling nodal fibroblastic reticular cells [28], and the endothelium differentiates into high endothelial venules, enabling continuous lymphocyte circulation from the bloodstream [29,30,31]. The establishment of distinct T- and B-cell zones results in the formation of TLSs, an organized structure that mirrors the architecture of the lymph node [32,33].

TLSs have been described in a variety of solid tumors, including those of the lung, breast, colon, and melanoma, where their presence generally correlates with favourable clinical outcomes [15,16,33].

TLSs represent a frequent histopathological feature of GC, although their reported prevalence varies considerably among published studies. Xie et al. observed the presence of TLSs in approximately 55% of GC cases [2], while Jiang et al. identified TLSs in as many as 95% of analyzed tumors [13]. Similarly, Zhang et al. detected that only 7.5% of resected GCs lacked both peritumoral and intratumoral TLSs, demonstrating a significant association with favorable survival outcomes [34]. In our cohort, TLSs were present in 66% of cases, placing our findings within the range of previously reported data. This wide range in the reported prevalence may reflect differences in patient population, tumor biology and biological differences within the TIM, and methodological approach used for TLS assessment.

Generally, our cohort exhibited a relatively short overall survival compared with previously reported European cohorts [35,36,37] (median 13.5 months). This likely reflects the high-risk profile of the included patients, who were all of older age (median 75 years) and predominantly presented with locally advanced disease, with stage III tumors accounting for 54.1% of cases.

In our cohort, patients with TLS-positive GCs had significantly better survival outcomes compared to patients with TLS-negative GCs, with the same trend observed across both histological subtypes. TLSs may support local activation and proliferation of lymphocytes within the tumor microenvironment. By enabling antigen presentation and priming of immune cells directly at the tumor site, TLSs reduce the need for lymphocyte recruitment from remote lymph nodes, allowing for an accelerated and effective cytotoxic response against tumor cells [26,27]. This locally organized immune activity may therefore explain the improved survival observed in patients with TLS-positive tumors. According to Zhang et al., the formation of TLSs predicts favorable lymphocytic infiltration and may serve as a biomarker for OS risk stratification [34]. In a spatial mapping analysis, Xie Y et al. demonstrated that TLSs, particularly those located in the tumor core, are associated with better prognosis and improved response to immune checkpoint blockade [2]. Meta-analyses and multicenter studies confirm these findings. Yu A et al. showed that the presence of TLSs is significantly associated with improved outcomes in gastrointestinal tumors [38]. However, the impact of TLSs on outcome depends on tumor type, location, and TLS maturation stage. According to the meta-analysis by Zao et al., intratumoral TLSs are associated with a better prognosis, while peritumoral TLS is favorable in some tumors (esophageal and colorectal cancer) and unfavorable in others (breast and liver cancer) [15]. Kemi N et al. did not confirm the association of the presence/density of TLSs in GC with OS in a multivariate analysis [4].

In our study, lymphovascular invasion and positive regional lymph nodes were more frequently observed in GCs with immature forms of TLSs, suggesting less effective local immune surveillance. In contrast, mature TLSs were larger, more numerous, and characterized by a higher density of FOXP3^+^ regulatory cells and a lower CD8^+^/FOXP3^+^ ratio, suggesting a more structured and functionally coordinated immune response. Comparable findings have been reported in other studies: higher maturity of TLS, usually defined by follicular organization, presence of germinal centers, and developed high endothelial venules, is associated with more favorable clinicopathological features and longer survival [15,39,40,41].

Furthermore, Posh et al. showed that a high proportion of mature TLSs is a stronger prognostic indicator than the total number of TLSs in non-metastatic colon cancer, which highlights the importance of TLS composition/maturation stage [42].

In line with previous reports, our analysis demonstrated significantly shorter overall survival in GCs containing only immature TLSs compared with tumors harboring mixed or fully mature TLSs.

Immature TLSs are often functionally inactive or even immunosuppressive, as they mainly contain FOXP3^+^ regulatory T cells and M2 macrophages that suppress cytotoxic response. Our finding of a correlation between immature TLSs with lymphovascular invasion and lymph node metastasis is consistent with previous reports [43,44]. While GCs with immature TLSs are typically enriched in immunosuppressive cell populations, mature TLSs contain abundant B cell clusters at various stages of differentiation, actively involved in antigen presentation and antibody production [3]. Moreover, plasma cells derived from TLSs have been shown to migrate into tumor nests, where they secrete antibodies directed against tumor antigens, thus contributing to the antitumor humoral immune response [45]. Meta-analyses across digestive system tumors confirm that only mature TLSs with germinal centers consistently confer survival benefit [38,46].

FOXP3^+^ regulatory T cells are essential for maintaining immune tolerance and preventing autoimmunity under physiological conditions. Within the tumor microenvironment, their function becomes detrimental. By suppressing cytotoxic activity and dampening antitumor immunity, TLSs enriched in regulatory T cells lose their usual protective role and may even promote tumor progression by creating a locally immunosuppressive milieu. As a result, cytotoxic immune activity is reduced, allowing tumor cells to persist, proliferate, and metastasize. In our series, TLS-positive tumors with FOXP3^+^ regulatory T cells present displayed higher mortality, supporting the notion that regulatory predominance within TLS may counteract their otherwise favorable prognostic effect. Previous studies have also linked high intratumoral FOXP3^+^ cells infiltration to poorer prognosis in GC [2,47]. According to Hennequin et al., in localized GC, marked infiltration of FOXP3^+^ cells within the tumor stroma was associated with shorter/reduced relapse-free survival (RFS) [48]. These results suggest that TLS confer prognostic advantage only when their internal structure supports effective immune activation. Immature or functionally inactive FOXP3^+^ rich TLSs fail to initiate an effective antitumor response, which contributes to faster disease progression and shorter patient survival.

In an exploratory analysis based on publicly available transcriptomic datasets, no statistically significant association was observed between FOXP3 mRNA expression and overall survival (*p* = 0.48). However, bulk transcriptomic analyses assess gene expression across the entire tumor tissue and therefore do not allow spatial localization of FOXP3^+^ immune cells. In contrast, the present study specifically evaluated FOXP3^+^ lymphocytes within tertiary lymphoid structures (TLS). This distinction is important because the biological and prognostic role of immune cells may depend on their spatial organization and functional context within the tumor microenvironment. TLS represent highly organized immune niches that resemble secondary lymphoid organs and provide a microenvironment for coordinated immune cell interactions [33]. Such spatial organization cannot be captured by bulk transcriptomic analyses [49,50]. Furthermore, mRNA expression levels do not necessarily correspond to protein expression levels due to post-transcriptional regulation [51]. Therefore, spatially resolved protein-level analyses, such as immunohistochemical assessment of FOXP3^+^ cells within TLS, may provide a more accurate understanding of their prognostic significance in gastric cancer.

The total number of CD8^+^ cells did not differ significantly, while, as expected, CD8^+^/FOXP3^+^ ratio was significantly higher in GCs with immature forms of TLSs, underscoring that the prognostic impact is determined not merely by cytotoxic T cell abundance, but by the interplay of effector and regulatory components within the TLS. These observations are consistent with the results of Hennequin et al., who analyzed survival outcomes of patients with GC, highlighting the importance of a coordinated Th1 and B cell response [48].

In summary, our findings suggest a trend toward improved clinical outcomes in patients with TLS-positive GCs and indicate a potential role of regulatory T cells in disease progression. However, these results should be interpreted with caution due to the relatively small sample size and the homogeneity of the studied population. Further large-scale and more rigorous studies are needed to validate our findings and to explore novel biomarkers in the context of the tumor microenvironment and contemporary therapeutic regimens.

## 5. Conclusions

The presence and maturation of TLSs in GC are associated with improved prognostic stratification. Assessment of TLS maturation and immune cell composition may provide valuable insights into disease progression. Further studies on larger and more diverse patient cohorts are warranted to elucidate the biological role of effector and regulatory T cells in the tumor immune microenvironment and their impact on clinical outcomes.

## Figures and Tables

**Table 1:** Clinicopathological characteristics according to the TLS status of GC.

Variable		TLS *n* (%) or Median (IQR)		*p*
		Present *n* = 66 (71.7%)	Not Present *n* = 26 (28.3%)	
Sex	male	46 (69.7)	17 (65.4)	0.689 *
	female	20 (30.3)	9 (34.6)	
Age, years		74.45 (62.5–81.25)	76 (67–80.25)	0.529 ^#^
Lauren subtype	intestinal	41 (62.1)	16 (66.7)	0.692 *
	diffuse/mixed	25 (37.9)	8 (33.3)	
Bormann type	polypoid	9 (13.6)	6 (24)	0.564 *
	ulcerative	24 (36.4)	6 (24)	
	ulcerovegetative	18 (27.3)	7 (28)	
	infiltrative	15 (22.7)	6 (24)	
pT	pT1, 2	16 (24.2)	3 (11.5)	0.175 *
	pT3, 4	50 (75.8)	23 (88.5)	
pN	negative	18 (27.3)	6 (23.1)	0.680 *
	positive	48 (72.7)	20 (76.9)	
Clinical stage	I, II	32 (49.2)	9 (34.6)	0.206 *
	III, IV	33 (50.8)	17 (65.4)	
Lymphovascular invasion	no	10 (20.4)	5 (23.8)	0.751 *
	yes	39 (79.6)	16 (76.2)	
Perineural invasion	no	20 (42.6)	6 (27.3)	0.222 *
	yes	27 (57.4)	16 (72.7)	
Overall survival, months		19.4 (6.5–42.1)	9.45 (2.23–15.05)	**0.006** ^#^

*—ꭓ^2^ test; # Mann–Whitney U test; bold indicates *p* < 0.05. TLS, tertiary lymphoid structures.

**Table 2:** Impact of TLS presence and clinicopathologic factors on overall survival in gastric cancer patients: Multivariable Cox regression.

Variable	HR (95% CI)	*p*-Value
Age	1.027 (1–1.055)	0.051
pT	1.258 (0.608–2.602)	0.536
pN	1.588 (0.82–3.077)	0.171
Lauren subtype	1.367 (0.771–2.421)	0.284
TLS status	0.497 (0.281–0.88)	**0.017**

Bold indicates *p* < 0.05.

**Table 3:** Impact of TLS maturation on clinicopathological features and immune composition in GC.

Variable		TLS *n* (%) or Median (IQR)		*p*
		Lymphoid Aggregate/Non-Mature *n* = 49 (74.2%)	Mature *n* = 17 (25.8%)	
Sex	Male	35 (71.4)	11 (64.7)	0.602 *
	Female	14 (28.6)	6 (35.3)	
Age, year		71.3 (59.5–80.3)	77 (67.5–82.5)	0.284 ^#^
Lauren subtype	Intestinal	31 (63.3)	10 (58.8)	0.745 *
	Diffuse/mixed	18 (36.7)	7 (41.2)	
Bormann classification	Polypoid	7 (14.3)	2 (11.8)	0.487 *
	Ulcerative	18 (36.7)	6 (35.3)	
	Ulcerovegetative	15 (30.6)	3 (17.6)	
	Infiltrative	9 (18.4)	6 (35.3)	
pT	pT1, 2	10 (20.4)	6 (35.3)	0.217 *
	pT3, 4	39 (79.6)	11 (64.7)	
pN	negative	10 (20.4)	8 (47.1)	**0.034** *
	positive	39 (79.6)	9 (52.9)	
Clinical stage	I, II	23 (47.9)	9 (52.9)	0.722 *
	III, IV	25 (52.1)	8 (47.1)	
Lymphovascular invasion	No	4 (11.4)	6 (42.9)	**0.014** *
	Yes	31 (88.6)	8 (57.1)	
Perineural invasion	No	14 (42.4)	6 (42.9)	0.978 *
	Yes	19 (57.6)	8 (57.1)	
Overall survival, months		15.2 (5.4–34)	34.55 (18.93–87.03)	**0.033** ^#^
TLS mean area, mm^2^		19 (9–125)	59 (19.25–190.75)	**0.016 ^#^**
Number of TLS **		4 (2–6)	8 (6–12.5)	**0.001 ^#^**
CD8^+^/mm^2^ of TLS **		750 (590–999)	790 (578.25–980.5)	0.795 ^#^
FOXP3^+^/mm^2^ of TLS **		27.27 (11.25–60)	169.05 (24.84–223.21)	**0.029 ^#^**
CD8^+^/FOXP3^+^		26.49 (19.73–73.3)	3.61 (3.3–28.37)	**0.013 ^#^**

*—ꭓ^2^ test; # Mann–Whitney U test; bold indicates *p* < 0.05. ** measured in three low-magnification fields. TLS, tertiary lymphoid structures.

**Table 4:** Impact of FOXP3^+^ regulatory T lymphocytes in TLSs and clinicopathologic factors on overall survival in gastric cancer patients: Multivariable Cox regression.

Variable	HR (95% CI)	*p*-Value
Age	1.059 (1.024–1.097)	**0.001**
pT	1.328 (0.527–3.345)	0.547
pN	2.061 (0.813–5.222)	0.127
FOXP3^+^ lymphocyte status	8.165 (3.341–19.955)	**<0.001**

Bolded *p* value < 0.05.

## Data Availability

The original contributions presented in this study are included in the article/Supplementary Material. Further inquiries can be directed to the corresponding authors.

## Supplementary Materials

The following supporting information can be downloaded at: https://www.mdpi.com/article/10.3390/medsci14010145/s1, Figure S1: Kaplan–Meier overall survival curves according to FOXP3 transcript levels in bulk tumor tissue. Table S1: Median overall survival (OS) times according to clinical stage.

## Abbreviations

The following abbreviations are used in this manuscript:

GC	Gastric cancer
TLS	Tertiary lymphoid structure
OS	Overall survival
TNM	Tumor-Node-Metastasis
WHO	World Health Organization
EBV	Epstein–Barr virus
HER2	Human epidermal growth factor receptor 2
SARIFA	Stroma AReactive Invasion Front Areas
TIM	Tumor immune microenvironment
FDCs	Follicular dendritic cells
HEVs	High endothelial venules
H&E	Hematoxylin and eosin
IHC	Immunohistochemistry
IQR	Interquartile range
RFS	Relapse-free survival
