# Supplementary material for: FOXP3+ Cells in Tertiary Lymphoid Structures Have Adverse Impact on Overall Survival in Patients with Gastric Cancer
Source: Med Sci (Basel). 2026 Mar 18;14(1):145. doi: 10.3390/medsci14010145 (PMC13027467; doi:10.3390/medsci14010145)
Supplement: Supplementary file 1 [file medsci-14-00145-s001.zip › medsci-4168991-supplementary/medsci-4168991-supplementary.pdf]

Supplementary Table S1. Median overall survival (OS) times according to clinical stage

Clinical stage (N, %)	OS, months (median, IQR)	p-value
I (9, 10.5%)	46.3 (11.7-91.8)	0.131*
II (27, 31.8%)	15.73 (2.93-34)	
III (46, 54.1%)	13.07 (5.53-30.27)	
IV (3, 3.5%)	10.8 (6.45-11.78)	

* Kruskal-Wallis H test

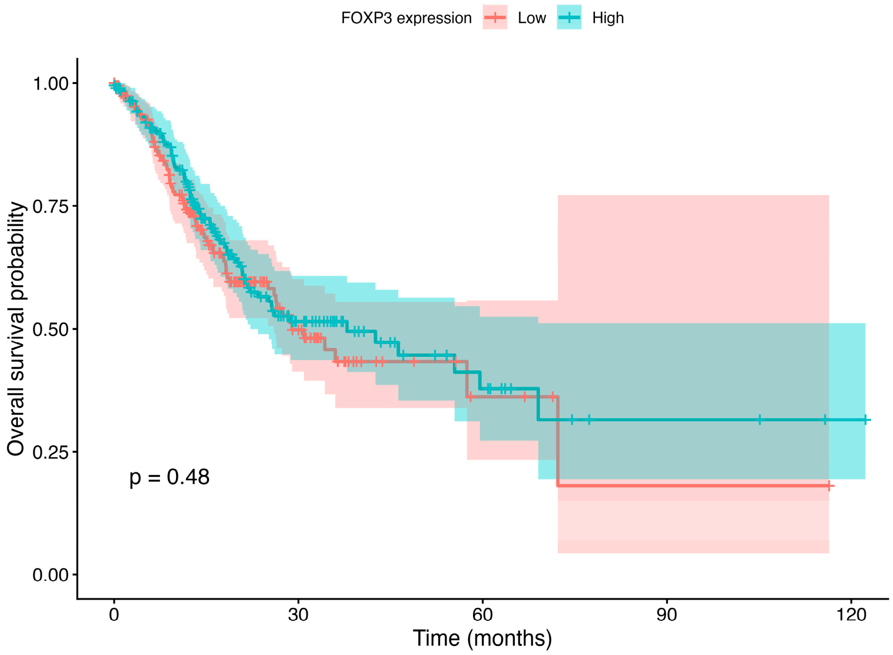

Supplementary Figure S1. Kaplan–Meier overall survival curves according to FOXP3 transcript levels in bulk tumor tissue.
